# Cultural and clinical challenges in sexual health care provision to men who have sex with men in Tanzania: a qualitative study of health professionals’ experiences and health students’ perspectives

**DOI:** 10.1186/s12889-021-10696-x

**Published:** 2021-04-07

**Authors:** Lucy R. Mgopa, B. R. Simon Rosser, Michael W. Ross, Gift Gadiel Lukumay, Inari Mohammed, Agnes F. Massae, Sebalda Leshabari, Ever Mkonyi, Stella Emmanuel Mushy, Dorkasi L. Mwakawanga, Maria Trent, James Wadley, Zobeida E. Bonilla

**Affiliations:** 1 Muhimbili University of Health and Allied Sciences (MUHAS), Dar es Salaam, Tanzania; 2 University of Minnesota, School of Public Health, 1300 S. 2nd St., Minneapolis, MN 55454 USA; 3 Johns Hopkins University, Washington, DC USA; 4 Lincoln University, Counseling and Human Services Department, 1570 Baltimore Pike, Philadelphia, PA 19352 USA

**Keywords:** MSM, Key population, Health care provider, Sexual health, HIV/AIDS

## Abstract

**Background:**

Health care providers across sub-Saharan Africa continue to face challenges while delivering sexual health care services. We explored the experiences, views and challenges of health care professionals and health students across different disciplines in Tanzania, towards delivery of sexual health services to men who have sex with men.

**Methods:**

Utilizing a qualitative approach, we recruited 121 health care professionals (providers) and students from the fields of midwifery, nursing and medicine in Dar es Salaam, Tanzania. We conducted 18 focus groups discussions, stratified by profession and experience, to investigate clinical management and challenges while addressing a case of an adult male presenting with rectal gonorrhea.

**Results:**

Findings indicated this case as extremely sensitive, clinical management involved establishing rapport and consent, medical care from history taking to treatment, and referral to other specialties. However, the illegal status of homosexuality in Tanzania was a primary concern to participants, this triggered the clinical care of this case scenario as challenging. There were uncertainties whether or not that such a case should be reported to the authorities.

**Conclusion:**

Findings from this study revealed a need for training health students in Tanzania to address sexual health issues including accurate information on homosexuality, reporting requirements and clinical management in the legal and socio-cultural context of the African continent.

## Background

One of the most important basic human needs is the ability of a person to have good health and easy access to quality health services. The World Health Organization (WHO) 2018 report emphasizes that poor quality of care is not only detrimental to humans but also contributes in diminishing resources needed to promote social and economic development that are important in human lives. The delivery of health care services can either be people-centered, focusing on community’s health or patient-centered, focusing on individuals’ seeking health care [1]. Human beings seek health care services when faced with physical illnesses, mental and psychological disorders as well as sexual health related problems. Sexual health incorporates sexual and reproductive health issues, sexually transmitted infections (STIs), HIV/AIDS, sexual violence, female genital mutilation, sexual orientation, gender identity and sexual dysfunction [2].

Sexual health is fundamental in human lives. It is deeply rooted in the sexuality and sexual behaviors of an individual across the life span, prevention and care of diseases and fulfillment of sexual relationships [3]. In developed countries, health care delivery systems strengthen access to sexual health services, promote sexual health education to both health care providers and patients, and encourage screening and prevention of HIV/STIs and related sexual problems [4, 5]. Furthermore, the delivery of these services is designed to target people of all genders, all age groups from adolescence on, and prioritize care to the most at-risk individuals including key populations [5].

“Key populations” is a term adopted by WHO, in common use across Africa, to denote five populations: men who have sex with men, people who inject drugs, transgender persons, sex workers and prisoners [6]. Key populations, including men who have sex with men (MSM), are those at highest risk of HIV, STIs, discrimination as well as victims of violence [7–9].

According to global estimates, more than one million sexually transmitted infections (STIs) are acquired every day. Twelve percent of these occur in the African region, followed by the regions of the Americas (8%) [10, 11]. Kirkcaldy et al. reported on the global prevalence of gonorrhea infection; MSM as a subgroup are disproportionately affected with rates close to 50% [12]. Sub-Saharan Africa remains the region most affected with HIV infections among all age groups. The highest HIV rates are in the most underdeveloped countries including Tanzania where the percentage of people living with HIV among adults of reproductive age was 5% based on 2018 data [13]. Prevalence estimates of HIV among MSM worldwide range between 3 to 25.4%, with 17.9% reported from sub-Saharan Africa [14]. Studies from Tanzania have reported significant rates of HIV and STIs among self-identified MSM from different regions, including HIV (11.1–30.2%), hepatitis B (5.4–8.0%), hepatitis C (3.4%), syphilis (0.2–2.5%) and herpes simplex virus 2 (38.5%) [15, 16]. Globally, sexual violence is also a major concern, estimated to be 7.2% directed towards women and 4.0% towards MSM [17, 18]. In sub-Saharan Africa, the rates of violence against MSM are extremely high [19, 20] with estimates from Tanzania exceeding 50% prevalence [18, 21, 22]. Homophobia towards MSM and transgender persons is astoundingly high on the African continent [23–25] with an overwhelming amount of stigma and hostility coming from society in general [22, 26]. Men who have sex with men are physically, sexually, psychologically and verbally abused, humiliated and compared to animals. As a former African president opined, “Homosexuals are worse than pigs and dogs”, exposed to negative health climates and as a result they end up facing biopsychosocial challenges [22, 25, 27–29]. Most African cultures are unfriendly towards MSM and other key populations. Hence, research focusing on the sexual health concerns among key populations is scarce compared to widely available data on heterosexual populations in Africa and countries like Tanzania [23, 24, 30–32].

### Sexual health care delivery

The provision of sexual health services should be integrated at every level of service from primary to tertiary health care, so that patients can have a chance to receive care from trained professionals. It is important for health care professionals (providers) of different disciplines to receive proper training in sexual health so that they are able to provide comprehensive care to patients. Such services include counselling, testing, advocacy, vaccination and treatment [33]. Despite providing primary healthcare, health workers in sub-Saharan Africa do not receive adequate training regarding sexual health. This leaves them vulnerable to having the same high levels of stigma and negative attitudes towards stigmatized groups, including sexual and gender minority populations and also young people who are sexually active. Health workers’ cultures and moral values, as well as lack of comfort in addressing sexual problems, are identified as common barriers to good clinical care [34–36].

By 2050, 40% of the world’s population is projected to reside in Africa [37], yet almost all research on homosexuality occurs outside of the continent. Across most of Africa, key populations face discrimination, judgements, and negative attitudes from health care workers. These factors act as barriers in accessing healthcare services by lesbian, gay, bisexual and transgender (LGBT) populations [27, 38, 39]. Furthermore, same sex relationships in sub-Saharan Africa are heavily stigmatized and criminalized in many countries in the continent [40, 41]. Two studies conducted in Eastern Africa revealed that MSM encounter several barriers towards accessing health care services due to their sexual orientation. These include being denied medications or provided with expired medications [27, 41], and in some instances, addressed as mentally ill patients or as “psychotic” [41, 42]. Because of the judgmental attitudes, discrimination, sociocultural and religious beliefs exerted by health care providers, MSM remain fearful in seeking health care services [27, 40–43]. This results in a continuous rise of HIV and STIs among this subpopulation in Africa as evidenced by a recent study conducted in Nigeria where the trend for HIV infection among MSM rose from 14 to 23% from the year 2007 to 2014 [44]. In addition, without training, health care providers shy away from initiating conversations about sexual orientation and/or sexual health with MSM clients, which leaves MSM clients/patients with limited or no information regarding their sexual health risks [27, 41, 42, 45, 46]. Comparisons across types of health facilities have shown that health care provision from private health facilities was considered more client-friendly towards both MSM and sex workers than care in public facilities [7, 27, 38, 42]. Younger health care providers were more welcoming and friendlier compared to health care providers who were much older and displayed a parental role to MSM patients, rather than a clinician’s role [42].

The purpose of this study was to conduct a socioecological needs assessment of sexual health care delivery in Tanzania. This was demonstrated by identifying, i) common sexual health problems seen in clinical practices, ii) barriers and facilitators when delivering sexual health services, iii) understanding the ability of local health delivery systems related to sexual health services, and iv) exploring cultural factors relevant to provision of sexual health care. The aim of this paper was to explore the experiences, views and challenges of health care professionals across disciplines and health students, towards delivery of sexual health services towards men who have sex with men.

## Methods

### Study design

This formative research is part of a larger mixed procedures research study for developing and evaluating an Afrocentric sexual health training program for midwifery, nursing and medical students attending Muhimbili University of Health and Allied Sciences in Dar es Salaam, Tanzania. We utilized a stratified 3 (profession: midwifery, nursing, medicine) by 2 (experience: (clinicians vs. students) design. The main objective was to assess and compare how providers address sexual health concerns across professions and by experience (providers versus students) in order to identify curriculum priorities. Focus groups were chosen because they are an efficient method to obtain information about clinical practices in a resource limited and conservative environment. This study was a collaboration between researchers at the University of Minnesota, USA and the Muhimbili University of Health and Allied Sciences (MUHAS), Tanzania. All methods were carried out in accordance with relevant university and national guidelines and regulations. The Institutional Review Boards of each institution and the National Institute of Medical Research (NIMR), Tanzania approved the ethical clearance and exempted the project from human subjects review since the focus was on clinical practice and development of the curriculum.

### Study participants

Participants were health care providers (i.e., nurses, midwives and medical doctors) with at least 2 years’ clinical experience recruited from three public and private hospitals. These were Muhimbili National Hospital, Mnazi Mmoja Hospital and Agakhan Hospital all located at Ilala District, Dar es Salaam. Stratified purposeful sampling was employed to recruit a sample diverse in characteristics and experience [47]. Sixty providers were recruited from fields of midwifery, nursing and medicine. The higher proportion of females in the sample, mirrors the national workforce distribution in the mentioned health cadres [48]. Participants recruited from medicine were from departments of pediatrics, family medicine, accidents and emergency, psychiatry and mental health and obstetrics and gynecology. If there was more than one provider who met eligibility criteria, we enrolled the provider with the most experience and expertise. The selection of providers with the most experience in this situation provided the team with information-rich participants, having frequent encounters with patients from diverse populations. This allowed better comparisons with the perspectives of students. The students enrolled were from midwifery, nursing and medicine in their fourth year (final year for nurses and midwives and pre-ultimate year for medical students). Sixty-one students were recruited using fliers and announcements placed around campus halls at MUHAS. Students interested in volunteering were asked to contact the study staff at MUHAS. Consistent with qualitative methods, we recruited a sample diverse in practical experience, gender and age to gain diverse opinions during the focus group sessions.

### Study procedures

**Fig. 1:**
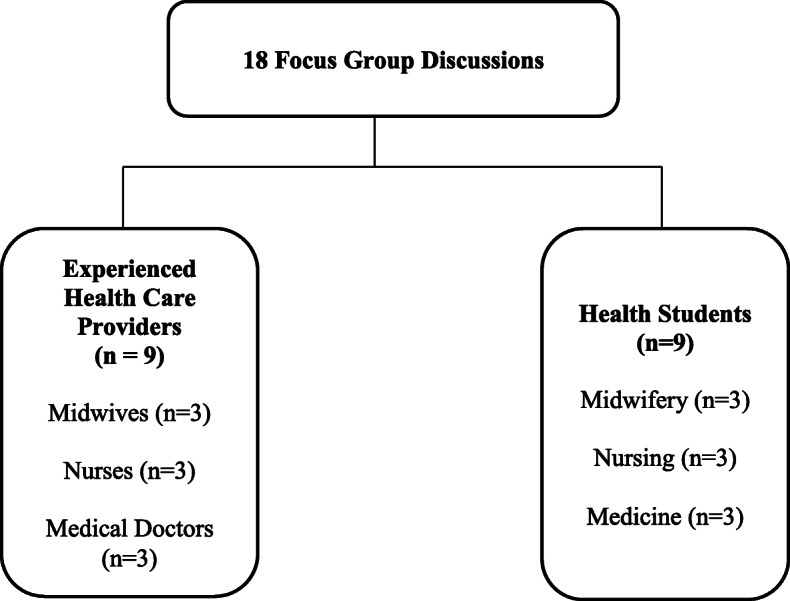
Focus Groups Study Design (Stratified by Profession and Experience)

In June, 2019, we conducted a total of 18 focus group discussions in a period of 1 month. Nine groups comprised of health care providers and the other nine were of health students. Each of the provider and student groups were further stratified by profession (midwifery, nursing and medicine; see Fig. 1**).** This stratification was to allow us to observe differences across professions and clinical experiences. Each group had an average of five to eight participants and the duration of the discussion was 60 to 90 min. To ensure confidentiality, the focus groups were held in convenient private rooms located within the hospital premises or at the university. The focus group moderator welcomed the participants and offered them refreshments to make them feel comfortable. Participants were provided with pen and paper to write their brief demographic characteristics (i.e. age, years of experience or clinical rotation, department) and a table card to write their first name or a nick name. Each group was led by a bilingual moderator assisted by a co-moderator who took notes and observed non-verbal expressions of the participants. While participants were told that they could discuss in English or Kiswahili whichever they preferred, the questions were posed in Kiswahili and the discussion was mostly in Kiswahili. A set of ground rules was shared in every focus group, to inform participants about their voluntary participation, rights to withdraw from the interview at any time, confidentiality and that there was an audio recorder recording the session. Participants received 60,000 Tanzanian Shillings (about US$25), to cover travel and other costs that they may have incurred.

The discussion was based on a semi-structured interview using a questionnaire that was developed specifically for this study. The interview guide started with an opening question about their current practice (for providers) or most recent clinical rotation (for students). Then, participants were asked to identify the most common sexual health challenges in Tanzania, and in their practice. These questions were followed by a series of clinical cases studies in which members of the groups were asked to give their opinions on how each case would get treated or not treated in Tanzania. Case presentations included male erectile dysfunction, wife beating, female dyspareunia and vaginal warts, a 14-year old girl requesting contraception, sexual abuse of a 9-year old male, male homosexual behavior, a rape victim, and finally, a case of adolescent masturbation. Following these, participants completed a separate survey about what they had received or not received training in, what they would prioritize in a sexual health curriculum, the formats for instruction, and comments on the curriculum. In this paper, we report the results from the clinical MSM case. For this case, the moderator read, “What about a man who has rectal gonorrhea? How would that be handled? Because homosexual behavior is stigmatized and illegal, a follow-up prompt asked, “And what would you write in the file in this case?

### Data management and analysis

At the end of each session, there was a 10–15 min debrief between moderator and co-moderator to discuss preliminary findings captured during the session, and any challenges the moderators encountered for the purpose of improving subsequent sessions. This written information was logged. The audio files were transcribed *verbatim*, then translated into English. Quantitative data, such as participant demographic characteristics, were entered into an Excel spreadsheet and analyzed. Transcripts were coded using inductive and deductive methods, as informed by grounded theory principles. A team-based coding approach for codebook development and thematic analysis was implemented and a step wise coding process during analysis was followed: 1) Codebook development; 2) Discovery of the themes, during open-ended coding; and 3) First coding cycle and axial coding and thereafter identification of categories and themes [49]. This approach allowed debates and intensive reflections of the data and generated a rich understanding of the participants’ contributions when delivering sexual health services.

#### Coding team

The six moderators who conducted the focus groups performed the data analysis. This team was composed of health professionals currently practicing in their respective fields and conducting clinical research and a doctoral student. The clinical and research experiences contributed by the team members enriched the data collection and analysis process, and it was resourceful during the phase of defining codes and data interpretations.

The codebook was initially developed through a list of deductive codes derived from the interview guide. Both deductive and inductive codes continued to be generated and were added to the codebook iteratively during the three steps of coding. Regular meetings between coders were conducted utilizing Google Docs for written feedback and constant updates regarding codes and definitions. Whenever there was an intercoder disagreement, it was resolved in a meeting of the whole team.

#### Discovery of themes

Open coding phase was conducted during an all team meeting at the University of Minnesota. The process involved multiple readings of three hard copy transcripts of the interviews and coding of the interviews manually. During this meeting, preliminary ideas and emerging themes were identified by the teams using an open-ended coding format without predetermined codes or categories. Pens, sticky notes, highlighters, and large flip charts were used to gather the early ideas and organize the codes under emerging themes. Each team had two coders and specific questions were assigned to the team based on expertise, background and interests. Each team manually coded the transcripts individually and then made a comparison with a team mate at the end of the open coding stage to assess agreements and reconcile differences. Each team needed at least 80% initial agreement. Codes generated during open coding were entered into the main codebook. During axial coding, each sub team generated larger categories and themes based on the findings of the first coding cycle. Codes were grouped under these larger findings of sub categories, categories and themes. Finally, the categories and themes were compared both between the professionals and the students, as well as across professions to identify any differences by experience and specialization.

## Results

As detailed in Table 1, we recruited a total of 121 participants. There were sixty experienced health care providers, including 11 men and 49 women. Their ages ranged from 24 years to 62 years, and had, on average, 15 years of clinical experience. Of the sixty-one students, 38 were men and 23 were women. Their age distribution ranged between 22 years to 37 years and all students were in their fourth year of their clinical programs. Female health care providers exceeded male providers across the three professions, while male students outnumbered their counterpart from schools of nursing and midwifery with exception from medical school. Overall, nursing and medical profession had forty participants each, whereas midwifery comprised a total of forty-one participants.

**Table 1:** Socio demographic data and types of focus group

	Midwifery	Nursing	Medicine	Total
**Sample Size**				
Students	20	19	22	61
Providers	21	21	18	60
	41	40	40	121
**Gender**				
Students	14 M; 6F	13 M; 6F	11 M; 11F	38 M; 23F
Providers	0 M; 21F	5 M; 16F	6 M; 12F	11 M; 49F
	14 M; 27F	18 M; 22F	17 M; 23F	49 M; 72F
**Age (in years)**				
Students: Mean	27.7	25.1	24.0	25.5
Range	23–37	23–27	22–28	23–37
Providers: Mean	44.5	41.1	43.5	43.1
Range	26–58	24–59	31–62	24–62
**Experience (in years)**				
Students	4	4	4	4
Providers: Mean	18.0	13.1	11.9	14.6
Range	4–38	2–30	4–25	2–38

From the analysis, two themes about the case study emerged: *Management of rectal STI in a man* which refers to the providers’ roles and obligations when taking care of the patient. The second theme reflected the providers’ *attitudes and dilemmas when managing a rectal STI.* This theme focused on the providers’ perceptions towards the patient and the difficulties they encounter while delivering these services (see Table 2).

**Table 2:** Themes, categories, sub-categories and selected codes that emerged during analysis

Themes	Categories	Sub-Categories	Selected codes
• Management of rectal STI in a man	• Treatment and follow up measures	• Establishing rapport	• Confidentiality • Assurance • Respect and openness • Provider and patient gender resemblance
		• Medical care, referral and documentation	• History taking • Physical examination • Investigation • Treatment • Psychological intervention • Social welfare support • GBV section • Written description of the patient case
• Attitude and dilemma when managing rectal STI	• Perception and barriers while delivering health care	• Labelling of the patient	• Homosexuality as an abnormal sexual behavior • Homosexuals referred as stupid men
		• Care provider’s confusions	• Unlawful practice • Provider’s uncertainty during care and treatment

### Theme 1: management of rectal STI in a man

The Focus Group Discussion (FGD) participants stated how they would handle a patient from the time he comes into contact with an individual caregiver to the point of referring him to other specialized professionals.

#### Treatment and follow up measures

##### Establishing rapport

Participants stated the first thing they would do is to establish a good rapport with a patient by being friendly, respectful, encouraging open communication between them and allowing the patient to feel comfortable by assuring him privacy and confidentiality. Considering that the patient is male with a sensitive complaint, some participants thought that there should be a consideration of gender resemblance between the provider and patient. These participants said they would ask if the patient was comfortable or not being attended to by a female healthcare provider.

*“I am thinking as a [manager] in charge of the clinic, first of all male patients have to be attended by a male. It is really difficult for a man to reveal things like this to a woman.” (FGD Respondent-Midwife student)*

##### Medical care, referral and documentation

Thereafter participants reported that a detailed history should be obtained with information about the patient’s identification, history of assault, risk behavior assessment (including history of having multiple sexual partners, safe sex practices including condom use, and transactional sex) and drug use history, together with medical and social history. A thorough physical examination would follow emphasizing the anal area with laboratory investigations that included HIV testing, and confirmatory tests for gonorrhea and other sexually transmitted infections. Participants mentioned that pre-test counseling for HIV testing would be part of this.

*“I will assure him of confidentiality so that he can give history … I will find out whether he was assaulted or has multiple partners and I will advise him. I will also find out the cause of whether he was assaulted and maybe he is doing that for income purpose.” (FGD Respondent – Medical Doctor)*

*“First, one must take proper history about his lifestyle, social life and his partners ... and source of his problem … also ask him if he is gay? Once found to have a disease then investigations are required after all tests. Then, you will know just what behaviors this person is involved in.” (FGD Respondent – Nurse Professional)*

In addition, more than half of respondents said they would provide medical treatment to the patient (if the confirmatory tests were positive). If the HIV test was positive, they would also consider the criteria for antiretroviral treatment initiation after counselling the patient. Next, they would advise the patient to bring his partner for testing, investigation and in the end treat the patient and his partner as a couple. Some participants also suggested psychological intervention if this was a case of sexual assault. And they thought about referring the patient to a psychologist. In most groups, participants stressed the importance of psychological intervention to help the patient to stop his behavior of having sex with men and practicing unsafe sex. Such counseling would include using condoms during intercourse and reducing the number of sex partners.

*“I think this is STI, so screening for HIV is important and talking about same sex and ask him to bring his partner for treatment … We will also advise him to stop doing that [having sex with men] and if he can’t, he needs to use condom just for HIV and STIs protection.” (FGD Respondent – Medical Doctor)*

*“When he has rectal gonorrhea, it means he conducts homosexual activities ... I will ask him if he was raped or not? Or if he is doing it willingly then I will treat his gonorrhea. I will also educate him about having one partner because changing to so many men will be easier for him to get gonorrhea.” (FGD Respondent-Medical student)*

Most participants reported that in order to have proper comprehensive care, a patient may need to be referred for professional help such as to a social worker, who will be expected to assess the patient’s social life and advise him accordingly. If this patient was a sexual assault victim, then the social worker would play a role in doing the social investigation and reaching out to the appropriate authorities such as the gender-based violence section within the hospital or at a nearby police station.

*“I will consider it as a gender-based violence case, so I will refer it to the GBV section [i.e., the office in the hospital that deals with gender-based violence].” (FGD Respondent- Nurse professional)*

*“Patient may have another problem which associate things like consulting psychiatry or social welfare.” (FGD Respondent – Medical student)*

Participants were probed about how they would document this case in the patient’s file. Most participants agreed that every patient’s file deserved a proper documentation in case a patient is referred to another specialist. Then, it would be easy for the subsequent care provider to note what has been previously done to the patient. With the exception of one student participant who reported that he would not write any revealing information about the patient’s complaints, the rest of the participants insisted they would write every detail from the clinical presentation, investigations, results and diagnosis. Some stressed that his homosexual behavior must be written in the same file. In addition, some participants stressed the need to provide the patient with education on STI risk reduction, but they did not want to give him condoms (in case it encouraged homosexual behavior).

*“I will write diagnosis, his sexual behavior and his treatment and everything the truth ... because for this kind of person looks like he is married [i.e. to a woman], then he has a man or men and this is a chain so I will also give him education but I will not give him condoms.” (FGD Respondent – Midwife professional)*

*“I will not write anything to show that this patient is gay. In fact, I will use medical terminologies to hide information to other people.” (FGD Respondent- Nurse student)*

### Theme 2: attitude and dilemma when managing rectal STI

#### Perception and barriers while delivering health care

##### Labelling of the patient

Despite the knowledge that STIs are highly prevalent in Tanzania, a significant proportion of participants defined the patient based solely on his clinical presentation with rectal gonorrhea. Some providers said that this patient was clearly practicing an abnormal sexual behavior or rather practicing unprotected anal sex. The participants went further in the discussion, referring to him as “homosexual”, “gay” and even more judgmentally, that he was a “stupid” man.

*“I attended a seminar in the past and they were 150-160 homosexuals. I was very shocked to meet these stupid men … When he has rectal gonorrhea then clearly, he is a person who conducts homosexual activities.” (FGD Respondent- Medical Doctor)*

*“Once you speak about rectal gonorrhea, that means this man is gay … I will ask him to reveal the truth that he is gay.” (FGD Respondent – Midwife student)*

##### Care provider’s confusions

Concerns about laws regarding same sex relationships in the country were raised by almost all participants. They stated that it is very challenging, yet confusing, to manage such a case and at the same time be expected to observe the laws and regulations of Tanzania. They pointed out that in some instances, a care provider may not know whether to treat or not to treat such a patient, or whether they are supposed to report him to the police based on the nature of his sexual practices. Others wondered aloud whether it was better to just be silent and do their jobs.

*“First point to note is that this person has gonorrhea because of anal sex. This, to me, is a big case and others will try to hide the truth ... I will observe him just to confirm he is gay. You know, those gays when you just look at them even their appearance you will know … This stuff in our country is illegal but maybe I will treat him… but I am in real dilemma situation but I think I will or not ... I do not know.” (FGD Respondent - Midwife student)*

*“Homosexuality is illegal in Tanzania so I am not sure whether after treating this man I am required to report [him to police]. I don’t know (sigh).” (FGD Respondent – Nurse student)*

Participants clearly explained how stressed they feel when such a patient seeks services. They stated they feared their job might be put in jeopardy just by being silent about the patient. Some participants stated a number of patients with this presentation might buy medications from pharmacies and treat themselves, rather than going to the hospital or clinic, because of the fear of being reported or imprisoned because of their sexual orientation.

*“Some, you may find they go to pharmacies and get medications because they are afraid, once too much, [if] he comes to the hospital.” (FGD Respondent- Midwife professional)*

Some (four) professionals had positive attitudes when caring for such patients based on their understanding of medical ethics. One professional stated that sexual reproductive and health care in general is a right to any person. Regardless of the situation or illness, they said that service should be given to all.

*“Rectal gonorrhea is among the STD’s ... and health care is a right to whoever comes for treatment regardless of the situation or kind of disease. I will talk to him and explain the effects of such behavior. If he thinks it’s fine to stop, it’s up to him. But ultimately, he has the right to be heard and to be treated.” (FGD Respondent – Midwife professional)*

### Comparisons between health professionals and students

The participants’ responses concerning management of this case scenario were similar across the three professions. But there were few interesting differences when we compared the students to the experienced providers. Under the first theme, participants approach to management was similar with regards to medical care up to referral, although in terms of *documentation,* one student stated they would obscure the man’s homosexuality in the file for the sake of preserving confidentiality. Based on the second theme, labelling of a patient as “homosexual,” “gay” or “stupid” based on his clinical presentation was similar across the groups, although professionals expressed more negative attitudes towards this patient when compared to the students. This could be due to individual cultural and moral values than abiding to medical ethics. Most participants stated their difficulties regarding providing care to a man having sex with other men was because of the existing laws and criminalization of same sex relationships in the country. However, fewer (four) health professionals than students believed in health care equality for all regardless of sex orientation or circumstances.

## Discussion

These results shed some very interesting light into how healthcare providers in Tanzania would approach such a patient. The key findings detailed: 1) the ways in which health care providers would provide sexual health services to a man who has had sex with another man; 2) perceptions of, and challenges faced by health care providers when encountered by MSM client or patient and 3) the differences in opinions and experiences between care providers in actual practice and those in training (students).

Participants in this study emphasized the importance of professionalism while treating the patient (MSM). They explicitly mentioned showing respect, keeping confidentiality, good communication, openness and, if possible, gender matching with a patient. These findings were similar to other two qualitative studies conducted in London by Howarth et al. [50] and from Kenya by Van der Elst and colleagues [51], that obtained and discussed similar aspects of professionalism when providing care to MSM. In addition, concerning record keeping, participants showed awareness regarding the danger of disclosure of the patient’s sexual information. Confidentiality was highly emphasized to be of utmost importance when documenting patient’s information. Health care providers need to be focused during documentation, include any confirmed diagnoses, and think carefully about what other details will be useful to write in the record. Nevertheless, clinicians are not mandated to report sexual orientation to any authority, and as in other countries clinicians are required to maintain confidentiality when treating a patient [38, 51, 52]. Despite this, deep stigma towards homosexuality, combined with lack of training in how to treat such patients, results in students and clinicians wondering about their professional responsibilities.

From the first theme, health providers and students showed several knowledge gaps. These included inaccurately framing homosexuality as a psychological disorder needing referral for treatment, and discussion of ways to dissuade the patient from having (homosexual) sex. A second myth included the belief that it is important to observe the patient to confirm, by his mannerisms, that he is MSM. A third was that the STI reflected the man’s promiscuity. A fourth more subtle myth was reflected in some providers seeing the patient as not necessarily important in his own right, but important as a vector of disease to heterosexual others (especially to a wife if he was married). And we highlight the tone of these discussions as pathologizing. The case was described by participants as “difficult,” “big,” with homosexual behavior likely the result of “violence.” These stand in contrast to care in the US and elsewhere, where the patient might be seen as normal and even responsible (by seeking treatment for STI symptoms). Notably absent was any discussion of reassuring the patient that homosexual orientation and behavior are normal variants of human behavior.

From the second theme, it appears that in the provision of health care services to MSM, the providers’ attitudes and perceptions towards MSM may reflect their socio-cultural background, as well as the political and legal status in Tanzania criminalizing same sex relations. These findings were similar to findings reported by Matovu et al. in a Ugandan study, who report that legislation affects access of health services by MSM and that health care providers report reservations in having MSM as patients due to personal attitudes and various sociocultural factors [38]. A study in Kenya found that religion, in addition to other sociocultural factors, influenced how providers perceive MSM. But in Kenya, the providers did not report concerns with their country’s laws regarding health service provision to MSM [53]. Our findings show that most of our participants did not have a clear knowledge of their obligations, when providing services to MSM. In a conservative country like Tanzania where MSM are both illegal and heavily stigmatized, the providers’ attitudes appear shaped by their background and wider society.

Although MSM and other key populations are victims of legal and sociocultural hostility in sub-Saharan Africa [28, 29, 54], health professionals caring for such populations are not exempted from the mentioned risks as referenced in our study and elsewhere [51]. Despite the scarcity of data depicting challenges facing health care providers servicing key populations in Tanzania, most of sub-Saharan African communities may appear judgmental towards these populations as well as their care providers [43, 53, 55]. Hence, legal and sociocultural restrictions may create dilemma among health care providers when providing services to key populations including MSM [51, 56].

Provision of health services to MSM is of utmost importance, given the disproportionate burden of HIV and STIs among this population. Similar to the conclusions in studies from South Africa, Uganda, and Malawi, we conclude that in Tanzania, MSM are as entitled as any other human beings to sexual health services. This is true, regardless of their sexual orientation or appearance and critical in order to promote good care and to prevent transmission of sexually transmitted illnesses [43, 52, 55].

In contrasting the students and experienced clinicians’ responses, the experienced providers expressed more negative attitudes but also more details on care. This likely reflects the students having less practical experience. Similar to findings from Ghana and Malawi, our findings show the need to train both experienced health care providers and students in sexual health, in order to address and minimize the judgmental and negative attitudes towards MSM expressed across the focus groups [43, 57].

Given the prevalence of homosexual behavior in the population, it is very likely for a health care provider to encounter a patient who reports same sex relations or behavior. Providers need training in their professional responsibilities when caring for these patients. Such training should reflect the highest ethical standards of providing care without prejudice to all in need, including to MSM. Such a training should also address how to provide care without discrimination, judgements and/or personal values being imposed on MSM patients. Advocating for user friendly services would promote disclosure of clinical symptoms (and sexual identity from sexual minority patients), thus improving the health status of MSM in general [27, 43]. Furthermore, when care providers are trained and experienced in addressing clinical problems concerning MSM, it promotes comfort and confidence in the provider when handling any sexual related concern raised by MSM or other key populations [43, 57].

Across sub-Saharan African, most health services are unfriendly to MSM, reflecting the broader sociocultural and political environment across the region. This situation is likely maintained by several factors. Chief among them is the lack of training opportunities for health care professionals in sexual health. Care providers should not only know their roles and obligations when providing care to MSM, they need to understand the health needs of MSM patients and appreciate the broader challenges faced by this population. We recommend that training in sexual health for African health students is needed and needs to be Afrocentric to address the health priorities on the continent, the attitudes expressed in our findings, and the legal and ethical concerns that emerge due to context. Moreover, such training should be extended to health care providers who are in current practice through seminars or continuous medical education (CME), so that they can also be equipped with knowledge and practical skills.

### Study limitations

Our findings must be considered in light of several study limitations. First, recruitment of the experienced health providers was limited to three hospitals located in Dar es Salaam, a metropolitan area and not from the rural areas (upcountry). Hence, our results may not generalize to health care providers working in other parts of the country or other regions in sub-Saharan Africa. Secondly, some providers, and in particular the midwives, could be expected to have less contact with MSM patients as compared to medical doctors and nurses. Therefore, the answers obtained from the midwives may have been driven more by personal beliefs and more based in what they thought they should do, rather than in actual clinical experience.

The results of this study are being used to inform a sexual health training curriculum tailored to address the sexual health concerns of health students training in Tanzania. Based in part on these results, a unit on sexual orientation will focus on the dual themes of providing competent care to sexual and gender diverse patients, while confronting negative attitudes of providers. In the next stage of this research, our team will pilot and evaluate a curriculum to midwifery, nursing and medical students that includes content on sexual orientation and gender identity.

## Conclusion

Health care providers and health students discussed in depth, how a case of rectal gonorrhea would be handled in Tanzania. There was a recognition that MSM patients deserve to receive sexual health services despite the prevailing restrictive laws and widespread social antipathy towards MSM. Providers and students clearly struggled with how to treat MSM, a situation they identified as made more difficult by the law criminalizing homosexual behavior. Widespread negative attitudes by providers and students towards this population were noted. The authors of this research study are respectful of the culture and laws in Tanzania without any prejudice, but rather focused in training and imparting knowledge to health professions students who are the country’s future health care providers. Additional research is warranted to assess the views of health care providers from other (rural) areas and capture the extent of their understanding and awareness of provision of care to MSM, as well as suggestions that may contribute to providing appropriate sexual health care to MSM.

## Data Availability

The qualitative transcripts generated in this study are not publicly available given that sexual health is a highly sensitive topic in Tanzania. The datasets generated and/or analysed during the current study are not publicly available due to confidentially assurances provided to the participants but are available from the corresponding author on reasonable request.

## Abbreviations

AIDS: Acquired Immunodeficiency Syndrome
HIV: Human immunodeficiency virus
LGBT: Lesbian, Gay, Bisexual and Transgender
MSM: Men who have sex with men
STI: Sexually transmitted illness

## References

[1] Delivering quality health services (2018). a global imperative for universal health coverage.

[2] Sexual health and its linkages to reproductive health (2017). An operational approach.

[3] Centers for Disease Control and Prevention (US) (2010). Division of Reproductive Health. Reproductive health: major milestones.

[4] Ford JV, Barnes R, Rompalo A, Hook EW (2013). Sexual Health Training and Education in the U.S. Public Health Rep.

[5] Developing sexual health programmes (2017). A framework for action.

[6] Key populations. Targeted approaches to an AIDS – free generation: USAID; 2019. www.usaid.gov/global-health/health-areas/hiv-and-aids/technical-areas/key-populations

[7] Duorado I, Crosland Guimaräes M, Damacena G, Magno L (2019). Sex work stigma and non-disclosure to health care providers: data from a large RDS study among FSW in Brazil. BMC Int Health Hum Rights.

[8] Consolidated guidelines on HIV prevention, diagnosis, treatment and care for key populations. World Health Organization (2014). http://www.who.int/hiv/pub/guidelines/keypopulations 25996019

[9] Evens E, Lanham M, Santi K, Cooke J, Ridgeway K, Morales G, Parker C, Brennan C, de Bruin M, Desrosiers PC, Diaz X, Drago M, McLean R, Mendizabal M, Davis D, Hershow RB, Dayton R (2019). Experiences of gender- based violence among female sex workers, men who have sex with men and transgender women in Latin America and the Caribbean: a qualitative study to inform HIV programming. BMC Int Health Hum Rights.

[10] Report on global sexually transmitted infection surveillance, 2018. Geneva: World Health Organization; 2018.

[11] Rowley J, Vander Hoorn S, Korenromp E, Low N, Unemo M, Abu-Raddad LJ, et al. Global and regional estimates of the prevalence and incidence of four curable sexually transmitted infections in 2016: WHO Bulletin; 2019. https://www.who.int/bulletin/online_first/BLT.18.228486.pd 10.1371/journal.pone.0143304 PMC4672879 26646541

[12] Kirkcaldy RD, Weston E, Segurado AC, Hughes G. Epidemiology of gonorrhea: a global perspective. Sex Health. 2019;16(5):401–11. 10.1071/SH19061. 10.1071/SH19061 PMC7064409 31505159

[13] Joint United Nations Programme on HIV/AIDS. UNAIDS (2018). 12349391

[14] Beyrer C, Baral SD, Griensven FV, Goodreau SM, Chariyalertsak S, Wirtz AL (2012). Global epidemiology of HIV infection in men who have sex with men. Lancet.

[15] Ross MW, Nyoni J, Ahaneku HO, Mbwambo J, McClelland RC, McCurdy SA (2014). High HIV seroprevalence, rectal STIs and risky sexual behavior in men who have sex with men in Dar es Salaam and Tanga, Tanzania. BMJ Open.

[16] Mmbaga EJ, Moen K, Makyao N, Mpembeni R, Leshabari MT. HIV and STI among men who have sex with men in Dodoma municipality, Tanzania: a cross-sectional study. Sex Transm Infect (2017); 0:1–6. Doi:10.1136/sextrans-2016-052770, 93, 5. 10.1136/sextrans-2016-052770 28202736

[17] Global and regional estimates of violence against women: prevalence and health effects of intimate partner violence and non-partner sexual violence. World Health Organization;2013.

[18] HIV and Young Men who have sex with men (2015). A Technical Brief: World Health Organization.

[19] Kaighobadi F, Collier K, Reddy V, Lane T, Sandfort T. Sexual violence experiences among black gay, bisexual and other men who have sex with men and transgender women in South African townships: contributing factors and implications for health. S Afr J Psychol. 2019. 10.1177/0081246319859449. 10.1177/0081246319859449 PMC7880497 33583966

[20] Miltz AR, Lampe FC, Bacchus LJ, McCormack S, Dunn D, White E, Rodger A, Phillips AN, Sherr L, Clarke A, McOwan A, Sullivan A, Gafos M (2019). Intimate partner violence, depression and sexual behavior among gay, bisexual and other men who have sex with men in the PROUD trial. BMC Public Health.

[21] Mgopa L, Mbwambo J, Likindikoki S, Pallangyo P (2017). Violence and depression among men who have sex with men in Tanzania. BMC Psychiatry.

[22] Anderson AA, Ross MW, Nyoni JE, McCurdy SA (2015). High prevalence of stigma -related abuse among a sample of men who have sex with men in Tanzania: Implications for HIV prevention. AIDS Care.

[23] Ross M, Nyoni J, Larsson M, Mbwambo J, Agardh A, Kashiha J, McCurdy S (2015). Health care in a homophobic climate: the SPEND model for providing sexual health services to men who have sex with men where their health and human rights are compromised. Glob Health Action.

[24] Ochonye B, Foyalan M, Fatusi A, Bello B, Ajidagba B, Emmanuel G, Umoh P, Yusuf A, Jaiyebo T (2019). Sexual practices, sexual behaviours and HIV risk profile of key populations in Nigeria. BMC Public Health.

[25] Lane T, Mogale T, Struthers H, McIntyre J, Kegeles S (2008). “They see you as a different thing”: the experiences of men who have sex with men with health Care Workers in South African Township Communities. Sex Transm Infect.

[26] Tsang Yuk-ha E, Qiao S, Wilkinson JS, Fung Lai-chu A, Lipeleke F, Li X (2019). Multilayered stigma and vulnerabilities for HIV infection and transmission: a qualitative study on male sex Workers in Zimbwabwe. Am J Mens Health.

[27] Magesa D, Mtui L, Abdul M, Kayange A, Chiduo R, Leshabari M, Kayombo E, Tungaraza D (2014). Barriers to men who have sex with men attending HIV related health services in Dar es Salaam, Tanzania. Tanzania J Health Res.

[28] Zahn R, Grosso A, Scheibe A, Bekker LG, Katende S, Dausab F, Lipinge S, Beyrer C (2016). Human rights violations among men who have sex with men in southern Africa: comparison between legal contexts. PLoS One.

[29] Semugoma P, Nemande S, Baral SD. The Irony of Homophobia in Africa. Lancet (2012); 380(9839):312–314. doi: 10.1016/S0140-6736(12)60901-5. 10.1016/S0140-6736(12)60901-5 22819661

[30] MMbaga E, Leyna G, Leshabari M, TersbØl, Lange T, Makyao N, Moen K, Meyrowitsch D (2019). Effectiveness of health care workers and peer engagement in promoting access to health services among population at higher risk for HIV in Tanzania (KPHEALTH): Study protocol for a quasi-experimental trial. BMC Health Serv Res.

[31] Nyblade L, Mbote D, Barker C, Morla J, Mwai D, Oneko T, Stockton M, Dutta A, Kimani J, Musyoki H, Njugana S, Sirengo M (2015). Impact of Stigma on Utilization of Health Services among Sex Workers in Kenya.

[32] Moen K, Aggleton P, Leshabari MT, Middleton AL. Same – Sex Practicing Men in Tanzania from 1860 to 2010. Arch Sex Behav. 2014. 10.1007/s10508-014-0286-2. 10.1007/s10508-014-0286-2 24752788

[33] Meek J, Dunnett K, Brittain D (2014). The role of the HCA in sexual health services: British Journal of Health Care Assistants.

[34] Müller A, Röhrs S, Hoffman-Wanderer Y, Moult K (2016). “You have to make a judgement call”. – Morals, judgements and the provision of quality sexual and reproductive health services for adolescents in South Africa: Social Science and Medicine.

[35] Ross MW, Leshabari S, Rosser BRS, Trent M, Mgopa L, Wadley J, Kohli N (2018). Evaluation of an assessment instrument for a sexual health curriculum for nurses and midwifery students in Tanzania: the sexual health education for professionals scale(SHEPS). Appl Nurs Res.

[36] Alli F, Maharaj P, Vawda M (2013). Interpersonal relations between health care workers and young clients: Barriers to accessing sexual and reproductive health care. J Community Health.

[37] United Nations Department of Economic and Social Affairs. World population prospects 2019: Highlights2020 [cited 2020 July 26]. Available from: https://www.un.org/development/desa/publications/world-population-prospects-2019-highlights.html.

[38] Matovu J, Musinguzi G, Kiguli J, Nuwaha F, Mujisha G, Musinguzi J (2019). Health providers’ experiences, perceptions and readiness to provide HIV services to men who have sex with men and female sex workers in Uganda- a qualitative study. BMC Infect Dis.

[39] Duby Z, Scheibe A, Nkosi B (2019). “We must treat them like all the other people”: Evaluating the Integrated Key Populations Sensitivity Training Programme for Healthcare Workers in South Africa. South Afr J HIV Med.

[40] Fay H, Baral SD, Trapence G, Motimedi F, Umar E, Lipinge S, Dausab F, Wirtz A, Beyrer C (2010). Stigma, health care access, and HIV knowledge among men who have sex with men in Malawi, Namibia and Botswana. AIDS Behav.

[41] King R, Sebyala Z, Ogwal M, Aluzimbi G, Apondi R, Reynolds S, et al. How men who have sex with men experience HIV health Services in Kampala. Uganda BMJ Global Health. 2020;5(4). 10.1136/bmjgh-2019-001901.

[42] Shangani S, Naanyu V, Operario D, Genberg B. Stigma and Healthcare – Seeking Practices of Men Who Have Sex with Men in Western Kenya: A Mixed -Methods Approach for Scale Validation. AIDS Patient Care STDs. 2018;32(11). 10.1089/apc.2018.0101. 10.1089/apc.2018.0101 PMC6247373 30398953

[43] Kapanda L, Jumbe V, Izugbara C, Muula AS (2019). Healthcare providers’ attitudes towards care for men who have sex with men (MSM) in Malawi. BMC Health Serv Res.

[44] Eluwa GI, Adebajo SB, Eluwa T, Ogbanufe O, Ilesanmi O, Nzelu C (2019). Rising HIV prevalence among men who have sex with men in Nigeria: a trend analysis. BMC Public Health.

[45] Fisher C, Fried A, Macapagal K, Mustanski B (2018). Patient- provider communication barriers and facilitators to HIV and STI preventive Services for Adolescent MSM. AIDS Behav.

[46] Rebe K, De Swardt G, Struthers H, McIntyre J (2013). Towards ‘men who have sex with men-appropriate’ health services in South Africa. S Afr J HIV Med.

[47] Palinkas LA, Horwitz SM, Green CA, Wisdom JP, Duan N, Hoagwood K (2015). Purposeful sampling for qualitative data collection and analysis in mixed method implementation research. Admin Pol Ment Health.

[48] Exavery A, Lutambi AM, Wilson N, Mubyazi GM, Pemba S, Mbaruku G (2013). Gender-based distributional skewness of the United Republic of Tanzania’s health workforce cadres: a cross-sectional health facility survey. Hum Resour Health.

[49] Saldaña J. The coding manual for qualitative researchers: Sage Publications Ltd; 2009.

[50] Howarth AR, Day S, Greene L, Ward H. “They made me feel comfortable”: a comparison of methods to measure patient experience in a sexual health clinic. BMC Health Serv Res. 2017;17(1):325. 10.1186/s12913-017-2264-6, “They made me feel comfortable”: a comparison of methods to measure patient experience in a sexual health clinic. 10.1186/s12913-017-2264-6 PMC5418717 28476173

[51] Van der Elst EM, Gichuru E, Omar A, Kanungi J, Duby Z, Midoun M, et al. Experiences of Kenyan healthcare workers providing services to men who have sex with men: qualitative findings from a sensitivity training programme. J Int AIDS Soc. 2013;16(4suppl 3):18741. 10.7448/IAS.16.4.18741. 10.7448/IAS.16.4.18741 PMC3852126 24321109

[52] Wanyeze RK, Musinguzi G, Matovu JK, Kiguli J, Nuwaha F, Mujisha G, Musinguzi J (2016). “If You Tell People That You Had Sex with a Fellow Man, It is Hard to Be Helped and Treated”: Barriers and Opportunities for Increasing Access to HIV Services among Men Who Have Sex with Men in Uganda. PLoS One.

[53] Taegtmeyer M, Davies A, Mwangome M, Van der Elst EM, Graham SM, Price MA, Sanders EJ (2013). Challenges in Providing Counselling to MSM in Highly Stigmatized Contexts: Results of a Qualitative Study from Kenya. PLoS One.

[54] Consolidated guidelines on HIV prevention, diagnosis, treatment and care for key populations. World Health Organization. 2016 update. 25996019

[55] Rispel LC, Metcalf CA, Cloete A, Moorman J, Reddy V. You become afraid to tell them that you are gay: Health service utilization by men who have sex with men in South African cities. J Public Health Policy. 2011:S137–51. 10.1057/jphp.2011.29 21730987

[56] Rosser BRS, Mgopa L, Leshabari S, Ross MW, Gadiel Lukumay G, Massae A, Mkonyi E, Mohammed I, Mushy SE, Mwakawanga D, Trent M, Wadley J (2020). Legal and ethical considerations in delivery of sexual health care in Tanzania. Afr J Health Nurs Midwifery.

[57] Kushwaha S, Lalani Y, Maina G, Ogunbanjo A, Wilton L, Agyarko PT, Adu-Sarkodie Y (2017). “But the moment they find out that you are MSM…”a qualitative investigation of HIV prevention experiences among men who have sex with men(MSM) in Ghana’s health care system. BMC Public Health.

